# Energy-Efficient Optimal Power Allocation for SWIPT Based IoT-Enabled Smart Meter

**DOI:** 10.3390/s21237857

**Published:** 2021-11-25

**Authors:** Zaki Masood, Ardiansyah, Yonghoon Choi

**Affiliations:** 1 Department of Electrical Engineering, Chonnam National University, Gwangju 61186, Korea; 186204@jnu.ac.kr; 2 Department of Artificial Intelligence Convergence, Chonnam National University, Gwangju 61186, Korea; ardi@ejnu.net

**Keywords:** distributed antenna system, energy efficiency, energy harvesting, internet of things, smart grid, wireless power transfer

## Abstract

This paper presents an internet of things (IoTs) enabled smart meter with energy-efficient simultaneous wireless information and power transfer (SWIPT) for the wireless powered smart grid communication network. The SWIPT technique with energy harvesting (EH) is an attractive solution for prolonging the battery life of ultra-low power devices. The motivation for energy efficiency (EE) maximization is to increase the efficient use of energy and improve the battery life of the IoT devices embedded in smart meter. In the system model, the smart meter is equipped with an IoT device, which implements the SWIPT technique in power splitting (PS) mode. This paper aims at the EE maximization and considers the orthogonal frequency division multiplexing distributed antenna system (OFDM-DAS) for the smart meters in the downlink with IoT enabled PS-SWIPT system. The EE maximization is a nonlinear and non-convex optimization problem. We propose an optimal power allocation algorithm for the non-convex EE maximization problem by the Lagrange method and proportional fairness to optimal power allocation among smart meters. The proposed algorithm shows a clear advantage, where total power consumption is considered in the EE maximization with energy constraints. Furthermore, EE vs. spectral efficiency (SE) tradeoff is investigated. The results of our algorithm reveal that EE improves with EH requirements.

## 1. Introduction

In recent years, there has been growing interest in smart grid technology to build a green and energy-efficient smart city. The smart grid is transforming the way of conventional power generation and renewable resources to distributed energy between utilities and consumers. Various solutions have been proposed for the efficient use of energy and for the reduced operational cost of a smart city [1,2,3,4,5]. Within the next few years, the smart energy meter is likely to become an important component in smart grid technology. In general terms, smart meters can be described as a way to obtain power consumption in more detail than conventional power meters. Smart meters allow two-way communications between the meter and the grid network, which reduce cost and increase reliability. The smart meters also coordinate bidirectional electricity trading in the smart grid. This bidirectional communication provides a smart meter to collect information and energy at the same time. Multiple approaches have been suggested for smart meters to be more intelligent and reliable [6,7,8,9,10]. Much work on the potential of smart grid communication has been investigated, among them also power-line communication (PLC) [5,11,12,13]. PLC carries data on a conductor simultaneously, which is used in power distribution to the consumers. A major draw back of using PLC is the current attenuation due to the high voltage lines. Another disadvantage is the noise generated by the power line which is far more than telephone lines. On the other hand, advanced metering infrastructure (AMI) system collects and analyzes energy consumption on-demand [14]. It forwards the collected data back to the utility for serval purposes such as load forecasting, monitoring, and billing.

The internet of things (IoT) is an open network to transport data over the internet without demanding human to computer interaction. Many studies have been published on IoT, which provides a platform to control smart appliance and sensor devices [15,16,17,18,19,20,21]. Bedi et al. [15] highlight the evolution of IoT in transforming electric power and energy systems (EPESs) into secure, reliable, and intelligent EPESs. The real-time co-simulation of demand response (DR) policies in the smart grids network is investigated in [18]. The report in [18] exhibits the internet-connected intelligent devices located at customer premises and the smart grid to collect energy information and send commands. Lee et al. [20] propose a new method to secure data transport in a cellular network from a cellular-connected IoT device to a host. It has been suggested that the use of IoT is reliable for the future smart grid communication [21]. This approach of [21] seems to be reliable due to the remote monitoring and efficient control of the power flow among connected devices in an electric grid. In general, the IoT infrastructure requires smart devices, which can harvest energy from the different sources to wireless nodes. The wireless energy transfer (WET) is one of the possible solutions, which can transfer energy to prolong the battery life of the IoT device. Many attempts [22,23,24] have been made with the purpose of wireless power transfer (WPT), which comprises inductive coupling and electromagnetic (EM) radiations. Furthermore, radio frequency (RF) signals carry energy, which can be used in wireless powered communication networks (WPCNs) [25], where wireless nodes can utilize the RF energy into the power. Therefore, wireless energy harvesting (EH) can be a promising solution, which converts the received RF into energy. As a result, EH can be useful to increase the battery life for the IoT device. In recent years [26,27,28,29], there has been considerable interest in simultaneous wireless information and power transfer (SWIPT), where the user equipment (UE) can harvest energy and transfer information at the same time.

This approach is well suited for energy constraint relay systems to improve energy efficiency (EE) and information transfer simultaneously. In the last few years, much more information on SWIPT has become available, where power splitting (PS) and time switching (TS) modes are investigated. In the PS mode, the SWIPT technique performs two functions: one for EH and the other for information decoding (ID). Chae et al. [30] report a new scheme for PS-SWIPT based IoT sensor networks, which reduces the transmit power under EH constraints. Additionally, PS-SWIPT operation has been widely studied with various multiple-input multiple-output (MIMO) channels and distributed antenna system (DAS) [31,32,33]. In [34], optimal energy cooperation policy has been investigated, which focuses on the advantage of using DAS in a smart grid system. The DAS spatially divides antenna nodes connected to a centralized processor via a physical medium and delivers wireless connectivity to a fixed area. It can be applied to indoors or outdoors in any communication network, where antenna elevations at or below the clutter level, and port connections compact. In [35], the downlink orthogonal frequency division multiple (OFDM) SWIPT system with multiple IoT devices is investigated. Specifically, the resource allocation problem to maximize the secrecy rate for OFDM access (OFDMA), and time division multiple access (TDMA) systems are investigated, in a full-duplex network. More recent evidence [36] reports the EE maximization in the DAS-based IoT network with SWIPT technology adopting an optimal power allocation scheme. Ariffin et al. [37] investigate real-time energy trading strategy, where beamforming technique in a downlink green cloud radio access network (C-RAN) with SWIPT is adopted. Furthermore, the authors of [38] perform multiuser resource allocation for OFDMA system with SWIPT in the PS mode.

Most studies have only focused on EE with SWIPT in conventional cellular communication networks. Various papers have been presented to solve this issue. In previous work [39], we consider the DAS with PS-SWIPT system, which maximizes EE by optimal power allocation for an IoT device, the solution based on the Lagrange method and KKT conditions. In contrast to the previous work, in this paper we consider multiple IoT-enabled smart meters and propose an optimal power allocation algorithm for a OFDM-DAS system with PS-SWIPT. This paper aims EE maximization in the OFDM-DAS system for smart grid, where the smart meters can harvest energy and transfer information with an IoT enabled SWIPT technology. Thus, this approach provides the energy-efficient power allocation scheme in a smart grid and improves the performance of each smart meter, which is equipped with SWIPT function. This method is chosen because it is one of the feasible ways to implement SWIPT in wireless powered smart grid communication networks. The main contributions of this paper are summarized as follows:

(1) This research deals with multiple IoT-enabled smart meters, where the non-convex EE maximization problem is transformed into a subtractive form and the solution is based on proportional fairness.
(2) This paper takes a new look at the EE objective function and considers three constraints, i.e., EH constraint, PS ratio at the energy harvester, and DAS transmit power.
(3) An optimal power allocation algorithm is proposed for the non-convex EE maximization problem by adopting nonlinear fractional programming and the Lagrangian method.

We believe that our work presented in this paper will promote EH technologies in wireless powered smart grid communication networks.

This paper is organized as follows: Section 2 gives a brief overview of IoT-based smart grid system model. In Section 3, we analyze a non-convex EE maximization problem. A new methodology is outlined in Section 3 and a power allocation algorithm is proposed. Simulation results are presented in Section 4, followed by conclusion in Section 5.

## 2. System Model

**Figure 1:**
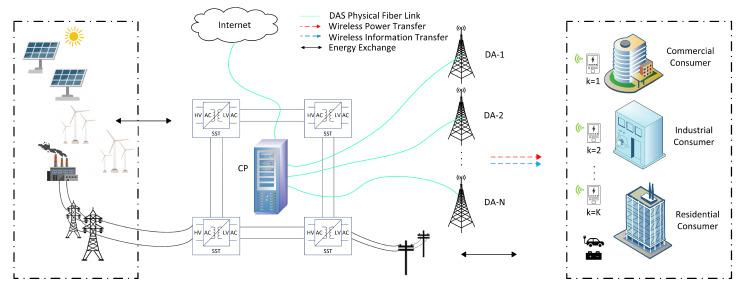
Structure of OFDM-DAS based IoT enabled PS-SWIPT in smart grid communication network.

As shown in Figure 1, wireless smart grid communication model can be classified in two layers: the smart grid layer and the communication layer. The renewable energy resources such as solar and wind are the self-generated power sources for the grid to satisfy the commercial, industrial and residential consumer load demands. In the smart grid layer, electric power flows from the distributed energy resources (DER) to the consumers. The DER generates electric power via conventional power plants or renewable sources, which are utilized to transport electricity to the electric load at the customer. On the other hand, in the communication layer, the consumer load is monitored by the smart meter. The essential part of the system in communication layer is a smart meter, which measures the power consumption of the home appliances and other devices. Here, it should be noted that in DAS all distributed antenna (DA) ports are connected via physical link to a central processor (CP), which performs DA port selection, DA power allocation, and subcarrier assignments. The useful data from the consumer devices are transferred transfer to the smart meter which enables IoT to exchange information via the Internet. Let us consider *K* smart meters and received signal at the *k*th smart meter is given by:

(1) $$\begin{array}{c} Y_{k}=hX_{k}+z, \end{array}$$

where h is the complex channel vector between DA port and smart meter, $X_{k}$ is the transmit signal from the DA port, and *z* indicates the additive Gaussian noise $CN(0,\sigma^{2})$ at the destination. In the system model, it is assumed that each smart meter is equipped with an IoT device, which implements SWIPT functions in the PS mode. Throughout this paper, we will use the term PS-SWIPT to refer to SWIPT operation in PS mode at the smart meter. For the PS-SWIPT function, $\rho_{k}$ denotes the splitting ratio. The information decodes with a part at the receiver is $\rho_{k}$, where the rest part $1-\rho_{k}$ for the EH part. Consequently, the two split signals at the destination for EH and ID, respectively.

**Figure 2:**
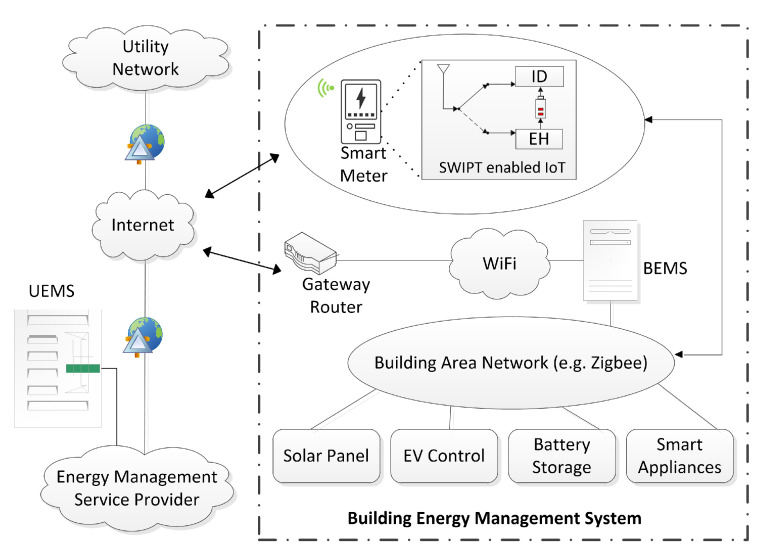
BEMS with IoT enabled PS-SWIPT, where smart meter in smart grid communication network exchange information between utility and BAN.

Figure 2 shows the building area network (BAN), where a local user intercommunicates own smart appliances, electric vehicle (EV) control, battery storage, and solar panel. Moreover, building energy management systems (BEMS) allow the user to monitor and automatically control the use of energy [40]. The BAN uses wireless technology based on Zigbee standards or home wiring over PLC. It can be used to remotely monitor or control the connected devices mentioned above. Furthermore, this management system connects a customer’s building network to the internet via WiFi connection. The smart meter interacts with the utility energy management system (UEMS) over the wireless communication network. We consider a downlink, OFDM-DAS system, where each DA port transmits data to the smart meter on multiple carrier frequencies. The bandwidth spectrum of this network is uniformly distributed into M subcarriers.

For information transmission among utility and smart grid, subcarriers can be allocated to each IoT device within smart meter. Moreover, each subcarrier allows only one user in the selected transmission time from the CP unit. Therefore, transmit power $p_{mnk}$ from DA port *n*, on subcarrier *m*, to the smart meter *k* is written as (2), where $P_{tot}^{max}$ denotes the total maximum transmit power. It is worth noting that each subcarrier is allocated to only a single IoT device in (3).

(2) $$\begin{array}{c} \;\sum_{k=1}^{K}\sum_{n=1}^{N}\sum_{m=1}^{M}p_{mnk}\leq P_{tot}^{max}, \end{array}$$

(3) $$\begin{array}{c} p_{mnk}\cdot p_{mnk^{{}^{\prime }}}=0,\;\forall m,n,k,\;k^{\prime }=\left\{1,2,\ldots ,K\right\},k^{\prime }\neq k, \end{array}$$

(4) $$\begin{array}{c} \;\sum_{k=1}^{K}\sum_{m=1}^{M}p_{mnk}\leq P_{n}^{max}, \end{array}$$

where m=1,2,…,M and n=1,2,…,N, which represents the *m*-th subcarrier and *n*-th DA port, respectively, and k=1,2,…,K is the smart meters equipped IoT enabled PS-SWIPT function. The maximum transmit power at the *n*-th DA port is $P_{n}^{max}$ in (4).

The acquisition of perfect CSI is available between DA port and IoT device, although it is practically challenging, however, there are techniques to obtain CSI for IoT devices [41]. It is assumed that the channel state information (CSI) is available between the DA port and IoT devices [42,43]. $h_{mnk}$ represents channel power gain between the IoT device and DA port. In the smart grid network, we consider only the downlink case for the coded information rate at the IoT device. The OFDM transfers the coded information on multiple subcarrier frequencies, which results in resilient to interference, and multipath effects. Additionally, it satisfies the spectral efficiency (SE) over the communication channel. It is well known that the information transmission rate $R_{k}$ of the received RF signal for ID receiver is given as

(5) $$\begin{array}{c} R_{k}\left(\rho_{k},p_{mnk}\right)=\frac{1}{M}\sum_{n=1}^{N}\sum_{m=1}^{M}\log_{2}\;\left(1+\frac{\rho_{k}h_{mnk}p_{mnk}}{\sigma^{2}}\right), \end{array}$$

where $\sigma^{2}$ is the Gaussian noise variance for the channel between IoT device and DA port, and *M* represents the total number of OFDM subcarriers.

It is important to note that, although the non-linear EH model is more practical [44], there are two reasons that we add the linear model, particularly into the PS-SWIPT system. The first reason is that the IoT-enabled smart meter has a low power regime for the SWIPT. Thus, it can be closely approximated by the linear EH model [45,46]. The second reason is that non-linear model is piecewise linear, where EH is easily trackable. Thus, for the EH part of the energy harvester, the harvested energy at the each IoT device can be written as:

(6) $$E_{k}\left(\rho_{k},p_{mnk}\right)=\xi \left(1-\rho_{k}\right)\sum_{n=1}^{N}\sum_{m=1}^{M}h_{mnk}\sum_{k^{{}^{\prime }}=1}^{K}p_{mnk^{{}^{\prime }}}.$$

Therefore, the received power at the *k*-th device is $\sum_{n=1}^{N}$ $\sum_{m=1}^{M}h_{mnk}\sum_{k^{{}^{\prime }}=1}^{K}p_{mnk^{{}^{\prime }}}$, where smart meter are equipped with IoT enabled PS-SWIPT function. In the relationship of EH, ξ is the energy conversion efficiency.

The RF-EH is considered as the dominant source in wireless green communication technology, as the demand for IoT and mobile base stations is increasing. Furthermore, from (6), it can be determined that an IoT device is able to decode information for its channel, and harvests energy from remaining channels. The efficiency of conversion adopting RF-EH is normally low, but energy is collected enough to recharge micropower devices such as IoT or remote sensors.

## 3. Problem Formulation

In the classical approach, the total power consumption is defined as (7), where μ is the reciprocal of the power amplifier drain efficiency, $P_{c}$ is the power conversion which represents power consumption into the circuit at the receiver. Thus, received power at the harvester can be written as:

(7) $$\begin{array}{c} \;P_{con}=\mu \sum_{k=1}^{K}\sum_{n=1}^{N}\sum_{m=1}^{M}p_{mnk}+P_{c}. \end{array}$$

Let us take advantage of utility function to model the modulation schemes in a power allocation problem. In our system model, IoT device with pre-specified application utility function can ensure optimal performance for a DAS system which outperforms regular schedulers. Unlike the conventional model, the power consumption in the SWIPT system can be compensated by the harvested power. This method is chosen because it is one of the most possible ways to include harvested energy into the system [47].

(8) $$\begin{array}{c} P_{total}\left(\rho_{k},p_{mnk}\right)=\mu \sum_{k=1}^{K}\sum_{n=1}^{N}\sum_{m=1}^{M}p_{mnk}+P_{c}-E_{k}\left(\rho_{k},p_{mnk}\right). \end{array}$$

Generally, EE can be defined as the ratio of total achievable transmission rate and the total power consumption. Therefore, EE of IoT enabled PS-SWIPT system can be written as:

(9) $$\eta_{EE}=\frac{\frac{1}{M}\sum_{k=1}^{K}\sum_{n=1}^{N}\sum_{m=1}^{M}\log_{2}\left(1+\frac{\rho_{k}h_{mnk}p_{mnk}}{\sigma^{2}}\right)}{\mu \sum_{k=1}^{K}\sum_{n=1}^{N}\sum_{m=1}^{M}p_{mnk}+P_{c}-\xi \left(1-\rho_{k}\right)\sum_{k=1}^{K}\sum_{n=1}^{N}\sum_{m=1}^{M}h_{mnk}.\sum_{k^{{}^{\prime }}=1}^{K}p_{mnk^{{}^{\prime }}}},$$

(10) $$\begin{array}{c} \;\left(P1\right):\;\;\;\;\;\max_{p_{mnk},\rho_{k}}\;\;\;\eta_{EE} \end{array}$$

(11) $$\begin{array}{c} \;\mathrm{subject}\;\mathrm{to}\;\;\;\;E_{k}\geq E_{min}, \end{array}$$

(12) $$\begin{array}{c} \;\sum_{k=1}^{K}\sum_{m=1}^{M}p_{mnk}\leq P_{n}^{max},\;\;\forall m,n, \end{array}$$

(13) $$\begin{array}{c} \;p_{mnk}\geq 0,\;\;\forall m,n, \end{array}$$

(14) $$\begin{array}{c} \;p_{mnk}\cdot p_{mnk^{{}^{\prime }}}=0,\;\forall m,n,k\neq k^{\prime }, \end{array}$$

(15) $$\begin{array}{c} \;0<\rho_{k}\leq 1. \end{array}$$

In the problem formulation, we consider the transmit power at the DA port, $E_{min}$, and PS ratio constraints (11)–(15). The constraint in (11) is the EH constraint, which limits the harvested power at the harvester with minimum EH requirements. The constraint in (12) is the transmit power constraint, which limits the transmit power at the DA port. This limits the $p_{mnk}$ to the peak transmit power $P_{n}^{max}$ at the DA port. The constraints (14) and (15) express the subcarrier allocation in the OFDM-DAS system, and limit the PS-SWIPT ratio for EH and ID function, respectively.

Since OFDM divides a channel into multiple subcarriers, let $\theta_{mnk}$ denote subcarrier assignment indicator for each IoT device. Thus, only one user is allowed on the same subcarrier for the proposed optimal power allocation. $\theta_{mnk}$ can have a value of 0 or 1. In other words, $\theta_{mnk}=1$ implies that subcarrier is allocated to the *k*-th user, otherwise $\theta_{mnk}=0$. Therefore, (P1) is modified with indicator constraint (19) and (20) as:

(16) $$\begin{array}{c} \;\left(P2\right):\;\;\;\;\;\max_{p_{mnk},\rho_{k}}\;\;\;\eta_{EE} \end{array}$$

(17) $$\begin{array}{c} \;\mathrm{subject}\;\mathrm{to}\;\;\;\;E_{k}\geq E_{min}, \end{array}$$

(18) $$\begin{array}{c} \;\sum_{k=1}^{K}\sum_{m=1}^{M}p_{mnk}\leq P_{n}^{max},\;\;\forall m,n, \end{array}$$

(19) $$\begin{array}{c} \;\sum_{k=1}^{K}\sum_{n=1}^{N}\theta_{mnk}=1,\;\;\forall m, \end{array}$$

(20) $$\begin{array}{c} \;\theta_{mnk}\in \left\{0,1\right\},\;\;\forall m,n,k. \end{array}$$

Next, we calculate the second order condition (SOC) of the information transmission rate of (5) and total power of (8) with respect to $p_{mnk}$. According to (21) and (22), $R_{k}$ is concave and $P_{total}$ is convex with respect to $p_{mnk}$. Therefore EE objective function with respect to $p_{mnk}$ is quasiconcave function.

(21) $$\begin{array}{c} \;\frac{\partial^{2}R_{k}\left(\rho_{k},p_{mnk}\right)}{\partial p_{mnk}^{2}}=-\frac{1}{Mln2}.\frac{\rho_{k}^{2}h_{mnk}^{2}}{{\left(\sigma^{2}+\rho_{k}h_{mnk}p_{mnk}\right)}^{2}}<0, \end{array}$$

(22) $$\begin{array}{c} \;\frac{\partial^{2}P_{total}\left(\rho_{k},p_{mnk}\right)}{\partial p_{mnk}^{2}}=0. \end{array}$$

In the case of (P2), the EE maximization problem is a quasi-concave for the power allocation variable $P_{n}^{max}$ [47]. Therefore, EE maximization problem (P2) with respect to constraints is a non-convex optimization problem. Next, we will propose an optimal power allocation scheme for the smart grid enabled PS-SWIPT in OFDMA-DAS system. We adopt proportional fairness to solve the EE maximization problem. The key benefit of this method is to achieve optimal power allocation while satisfying the minimum EH requirements. In addition to (P2), another constraint is expressed in (25), where $\theta_{mni}$ and $\theta_{mnj}$ are the set of predetermined values for proportional rate constraint between the number of smart meters on DA port. Therefore, (P2) is transformed to a new optimization problem in its equivalent subtractive form, which is formulated as (P3). Similar to [48], we adopt subcarrier allocation for the DAS-OFDM system, where $\Omega_{n}$ is the set of a subcarrier for a transmission rate of the *i*-th smart meter on DA port. It is important to note that EE objective function (P2) and (P3) are equivalent if and only if $F(\omega^{*})=0$ and $f\left(\omega^{*}\right)=p^{*}$ [49].

(23) $$\begin{array}{c} \begin{array}{cc} \left(P3\right):\;\max_{p_{mnk},\rho_{k}}\;\;\sum_{k=1}^{K}\sum_{m\in \Omega_{n}}\frac{1}{M}\log_{2}\left(1+\frac{\rho_{k}h_{mnk}p_{mnk}}{\sigma^{2}}\right)\\ \; & \;-\omega .\left[\mu \sum_{k=1}^{K}\sum_{m\in \Omega_{n}}p_{mnk}+P_{c}\right.\left.-\xi \left(1-\rho_{k}\right)\sum_{k=1}^{K}\sum_{n=1}^{N}\sum_{m=1}^{M}h_{mnk}\sum_{k^{{}^{\prime }}=1}^{K}p_{mnk^{{}^{\prime }}}\right] \end{array} \end{array}$$

(24) $$\begin{array}{c} \;\mathrm{subject}\;\mathrm{to}\;\;\;\;\;\;\mathrm{Equations}\;(17)\;\mathrm{to}\;(20), \end{array}$$

(25) $$\begin{array}{c} \;\frac{R_{i}}{R_{j}}=\frac{\sum_{m=1}^{M}\theta_{mni}}{\sum_{m=1}^{M}\theta_{mnj}},\;\forall i,j=\left\{1,2,\ldots ,K\right\},i\neq j. \end{array}$$

The function EE is a multivariable function and subject to constraints (17)–(20). Lagrangian function for EE maximization problem (P3) is given in (26), where $\lambda_{1,k},\lambda_{2}$ and $\lambda_{3,k}$ are the Lagrange multipliers for the constraints, respectively, ∀k. The Lagrangian function for the EE objective function can be written as:

(26) $$\begin{array}{c} \begin{array}{cc} \Lambda \left(p_{mnk},\rho_{k},\lambda_{1,k},\lambda_{2},\lambda_{3,k}\right)= & \sum_{k=1}^{K}\sum_{m\in \Omega_{n}}\frac{1}{M}\log_{2}\left(1+\frac{\rho_{k}h_{mnk}p_{mnk}}{\sigma^{2}}\right)-\omega .\left[\mu \sum_{k=1}^{K}\sum_{m\in \Omega_{n}}p_{mnk}+P_{c}\right.\\ - & \left.\xi \left(1-\rho_{k}\right)\sum_{k=1}^{K}\sum_{n=1}^{N}\sum_{m=1}^{M}h_{mnk}\sum_{k^{{}^{\prime }}=1}^{K}p_{mnk^{{}^{\prime }}}\right]+\sum_{k=1}^{K}\lambda_{1,k}\;\left(E_{k}-E_{min}\right)\;+\\ & \lambda_{2}\;\left(P_{m,n}^{max}-\sum_{k=1}^{K}\sum_{m\in \Omega_{n}}p_{mnk}\right)+\;\sum_{k=2}^{K}\lambda_{3,k}\left[\frac{1}{M}\sum_{m\in \Omega_{n}}\log_{2}\left(1+\frac{\rho_{1}h_{mn1}p_{mn1}}{\sigma^{2}}\right)-\right.\\ & \left.\frac{\sum_{m=1}^{M}\theta_{mni}}{\sum_{m=1}^{M}\theta_{mnj}}\frac{1}{M}\sum_{m\in \Omega_{n}}\log_{2}\left(1+\frac{\rho_{k}h_{mnk}p_{mnk}}{\sigma^{2}}\right)\right]. \end{array} \end{array}$$

Next, the optimal power allocation for the EE objective function can be obtained by partial derivatives of the Lagrange function with respect to $p_{mnk}$. The first order partial derivative condition for $p_{mnk}$ with Lagrange function can be written as:

(27) $$\begin{array}{c} \begin{array}{cc} \frac{\partial \left(p_{mnk},\rho_{k},\lambda_{1,k},\lambda_{2},\lambda_{3,k}\right)}{\partial p_{mn1}}= & -\omega \mu +\xi \left(1-\rho_{1}\right)\;h_{mn1}\;\left(\;\sum_{k^{{}^{\prime }}=1}^{K}\lambda_{1,k}+\omega \right)-\lambda_{2}\\ & +\frac{\rho_{1}h_{mn1}}{\left(\sigma^{2}+\rho_{1}h_{mn1}p_{mn1}\right)}\;\left(\frac{1}{Mln2}+\frac{1}{Mln2}\sum_{k=2}^{K}\lambda_{3,k}\right), \end{array} \end{array}$$

(28) $$\begin{array}{c} \begin{array}{cc} \;\frac{\partial \left(p_{mnk},\rho_{k},\lambda_{1,k},\lambda_{2},\lambda_{3,k}\right)}{\partial p_{mnk}}= & -\;\omega \mu +\xi \;\left(1-\rho_{k}\right)h_{mnk}\;\left(\sum_{k=1}^{K}\lambda_{1,k}+\omega \right)-\lambda_{2}+\\ & \frac{\rho_{k}h_{mnk}}{\left(\sigma^{2}+\rho_{k}h_{mnk}p_{mnk}\right)}\;.\;\left(\frac{1}{Mln2}-\frac{\sum_{m=1}^{M}\theta_{mni}\lambda_{3,k}}{\sum_{m=1}^{M}\theta_{mnj}Mln2}\right). \end{array} \end{array}$$

Thus, from (27) and (28), we set the partial derivative equal to 0 and obtain optimal power as:

(29) $$\begin{array}{c} \;\tau_{1}^{*}=\frac{1}{\left[\omega \mu -\xi \left(1-\rho_{1}\right)h_{mn1}\left(\omega +\sum_{k=1}^{K}\lambda_{1,k}\right)+\lambda_{2}\right]}.\left(\frac{1}{Mln2}+\frac{1}{Mln2}\sum_{k=2}^{K}\lambda_{3,k}\right)-\frac{\sigma^{2}}{\rho_{1}h_{mn1}} \end{array}$$

(30) $$\begin{array}{c} \;\tau_{2}^{*}=\frac{1}{\left[\omega \mu -\xi \left(1-\rho_{k}\right)h_{mnk}\left(1+\sum_{k=1}^{K}\lambda_{1,k}\right)+\lambda_{2}\right]}.\left(\frac{1}{Mln2}-\frac{\sum_{m=1}^{M}\theta_{mni}\lambda_{3,k}}{\sum_{m=1}^{M}\theta_{mnj}Mln2}\right)-\frac{\sigma^{2}}{\rho_{k}h_{mnk}} \end{array}$$

where $\lambda_{1,k}^{\left(i+1\right)},\lambda_{2}^{\left(i+1\right)}$ and $\lambda_{3,k}^{\left(i+1\right)}$ update the Lagrange multipliers. In order to obtain the optimal solution, we use the gradient method to update the Lagrange multipliers. Therefore, $\alpha_{1}^{\left(i\right)}$, $\alpha_{2}^{\left(i\right)}$ and $\alpha_{3}^{\left(i\right)}$ are the step size of the multipliers. Thus, multipliers can be written as:

(31) $$\begin{array}{c} \;\lambda_{1,k}^{\left(i+1\right)}={\left[\lambda_{1,k}^{\left(i\right)}+\alpha_{1}^{\left(i\right)}\left(E_{min}-E_{k}\right)\right]}^{+}, \end{array}$$

(32) $$\begin{array}{c} \;\lambda_{2}^{\left(i+1\right)}={\left[\lambda_{2}^{\left(i+1\right)}+\alpha_{2}^{\left(i\right)}\left(P_{m,n}^{max}-\sum_{k=1}^{K}\sum_{m\in \Omega_{n}}p_{mnk}\right)\right]}^{+}, \end{array}$$

(33) $$\begin{array}{c} \begin{array}{cc} \; & \;\lambda_{3,k}^{\left(i+1\right)}=\lambda_{3,k}^{\left(i+1\right)}+\alpha_{3}^{\left(i\right)}\left[\frac{1}{M}\sum_{m\in \Omega_{n}}\log_{2}\left(1+\frac{\rho_{1}h_{mn1}p_{mn1}}{\sigma^{2}}\right)-\right.{\left.\frac{\sum_{m=1}^{M}\theta_{mni}}{\sum_{m=1}^{M}\theta_{mnj}}\frac{1}{M}\sum_{m\in \Omega_{n}}\log_{2}\left(1+\frac{\rho_{k}h_{mnk}p_{mnk}}{\sigma^{2}}\right)\right]}^{+}. \end{array} \end{array}$$

Finally, optimal power allocation for the EE maximization problem can be written as:

(34) $$\begin{array}{c} \;p_{mn1}^{*}=min\left\{\tau_{1}^{*},P_{n}^{max}\right\}, \end{array}$$

(35) $$\begin{array}{c} \;p_{mnk}^{*}=min\left\{\tau_{2}^{*},P_{n}^{max}\right\}. \end{array}$$

In order to obtain the optimal solution for PS splitting ratio, similarly, we solve the partial derivatives for PS ratio, which can be written as:

(36) $$\begin{array}{c} \begin{array}{cc} \;\frac{\partial \Lambda \left(p_{mnk},\rho_{k},\lambda_{1,k},\lambda_{2,1},\lambda_{3,k}\right)}{\partial \rho_{1}}= & -\;\xi \left(\omega +\sum_{k=1}^{K}\lambda_{1,k}\right)\;h_{mn1}P_{mn1^{{}^{\prime }}}+\\ & \left(\frac{1}{Mln2}+\frac{1}{Mln2}\sum_{k=2}^{K}\lambda_{3,k}\right)\left(\frac{h_{mn1}p_{mn1}}{\sigma^{2}+\rho_{k}h_{mn1}p_{mn1}}\right), \end{array} \end{array}$$

(37) $$\begin{array}{c} \begin{array}{cc} \;\frac{\partial \Lambda \left(p_{mnk},\rho_{k},\lambda_{1,k},\lambda_{2,1},\lambda_{3,k}\right)}{\partial \rho_{k}}= & -\;\xi \;\left(\omega +\sum_{k=1}^{K}\lambda_{1,k}\right)\sum_{m\in \Omega_{n}}h_{mnk}P_{mnk^{{}^{\prime }}}+\\ & \left(\frac{1}{Mln2}+\frac{\theta_{k}}{\theta_{k^{{}^{\prime }}}}\frac{1}{Mln2}\sum_{k=2}^{K}\lambda_{3,k}\right)\;\left(\frac{h_{mnk}p_{mnk}}{\sigma^{2}+\rho_{k}h_{mnk}p_{mnk}}\right), \end{array} \end{array}$$

(38) $$\begin{array}{c} \begin{array}{c} \;\rho_{1}^{*}=\frac{\left(\frac{1}{Mln2}+\frac{1}{Mln2}\sum_{k=2}^{K}\lambda_{3,k}\right)}{\sigma^{2}+h_{mn1}p_{mn1}}\cdot \frac{h_{mn1}p_{mn1}}{\xi \;\left(\omega +\sum_{k=1}^{K}\lambda_{1,k}\right)\;h_{mn1}P_{mn1^{{}^{\prime }}}}, \end{array} \end{array}$$

(39) $$\begin{array}{c} \;\rho_{k}^{*}=\frac{\left(\frac{1}{Mln2}+\frac{\theta_{mni}}{\theta_{mnj}}\frac{\sum_{k=2}^{K}\lambda_{3,k}}{Mln2}\right)}{\sigma^{2}+h_{mnk}p_{mnk}}\cdot \frac{h_{mnk}p_{mnk}}{\xi \;\left(\omega +\sum_{k=1}^{K}\lambda_{1,k}\right)\sum_{m\in \Omega_{n}}h_{mnk}P_{mnk^{{}^{\prime }}}}. \end{array}$$

First, subcarrier is assigned to each DA port, and later remaining subcarriers are assigned in such a way that it maximizes the overall SE of the OFDM-DAS. Similar to [50], we adopt the subcarrier allocation to find the optimal ratio for $\theta_{mni}$ and $\theta_{mnj}$ constraints in (25), with rate constraint $\phi_{k}$ in Table 1. The optimal power allocation and PS-SWIPT ratio for EE maximization are summarized as Algorithm 1. The convergence of the proposed algorithm can be observed from the number of iterations in the inner and outer loops. Each iteration step in the inner loop finds an optimal transmit power for the given parameters in Table 2. Since (26) is a non-convex function, the closed-form solution is computationally challenging. Therefore, after $\lambda_{1,k},\lambda_{2}$ and $\lambda_{3,k}$ is set to fixed in the inner loop, optimal solution for $p_{mnk}^{*}$ can be obtained. Next, we adopt the gradient method to compute the Lagrange multipliers, which are small step sizes $\alpha_{1}^{\left(i\right)}$, $\alpha_{2}^{\left(i\right)}$ and $\alpha_{3}^{\left(i\right)}$ for updating [48].
**Algorithm 1:** Optimal transmit power for EE.1: **Initialization:** $\eta_{EE}=0$, ω=0.01, ζ=0.0005, i=02: Set channel gain $h_{mnk}$,  ∀m,n,k3: **for** n = 1 : N **do**4: **while**$\left(F\left(\omega \right)\geq \zeta \;\mathrm{and}\;\left|\lambda_{1,k}^{\left(i+1\right)}-\lambda_{1,k}^{\left(i\right)}\right|<\zeta \;\mathrm{and}\;\left|\lambda_{2}^{\left(i+2\right)}-\lambda_{2}^{\left(i\right)}\right|<\zeta \;\mathrm{and}\;\left|\lambda_{3,k}^{\left(i+3\right)}-\lambda_{3,k}^{\left(i\right)}\right|<\zeta \right)$5:    **for** k = 1 : K **do**6:        **for** m = 1 : M **do**7:            **if** k=1 **then**,8:               Solve $\tau_{1}^{*}$, obtain solution $p_{mn1}^{*}$ (29),9:            **else**10:             Solve $\tau_{2}^{*}$, obtain solution $p_{mnk}^{*}$ (30).11:       **end for**12:    **end for**13: Update $\lambda_{1,k}^{\left(i+1\right)},\lambda_{2}^{\left(i+1\right)}$ and $\lambda_{3,k}^{\left(i+1\right)}$ for i=i+1 by using (31), (32) and (33), respectively.14: **end while**15: Compute PS ratio $\rho_{1}^{*}$ and $\rho_{k}^{*}$16: By using $p_{mn1}^{*}$, $p_{mnk}^{*}$, $\rho_{1}^{*}$ and $\rho_{k}^{*}$, calculate optimal value of objective function $\eta_{EE}^{*}$17: **end for**

## 4. Numerical Results

In this section, simulation results are provided to validate the performance of the proposed algorithm for EE maximization. The results demonstrate the effectiveness and convergence of the proposed power allocation algorithm. In the simulations, we set the total number of DA ports and smart meter enabled IoT devices N=5 and K=15, respectively. The number of subcarriers that confirms our finding set to M=64.

Commonly, DAS is implemented as a cell, therefore in the simulation, it is considered that the DAS has a single cell structure in the smart grid network. Thus, main CP is fixed at the center and the number of DA ports is uniformly distributed in a cell. We assume that the DA ports are uniformly distributed in the smart grid communication network and located with polar coordinates $\left(\sqrt{\frac{3}{7}}R,\frac{2\pi (n-1)}{N}\right)$, where R=1000 m is the radius of the cell. For the numerical analysis, simulation parameters are considered according to Table 2. Therefore, for the N=5 DA ports, the location of *n*-th DA port can be determined in the OFDM-DAS based IoT-enabled smart grid network. The drain efficiency of the power amplifier μ is set to 0.38, whereas the energy conversion efficiency ξ at the IoT device is 0.6. The log-distance path loss model is adopted, and the path loss exponent is 3.7 dB. For PS-SWIPT operation, the splitting ratio $\rho_{k}$ at each IoT device ranges between 0 and 1. The shadow fading adopted as 8 dB, and noise power is set to −104 dBm.

For results comparisons, existing work shows EE maximization for DAS-SWIPT and OFDM-SWIPT, which have been published in [38,51,52,53]. A comparative table of these existing articles is listed in Table 3. First, we investigate the performance of EE and SE versus the number of iteration for the proposed power allocation algorithm. The relationship can be observed against the proportional fairness index $\phi_{k}$.

**Figure 3:**
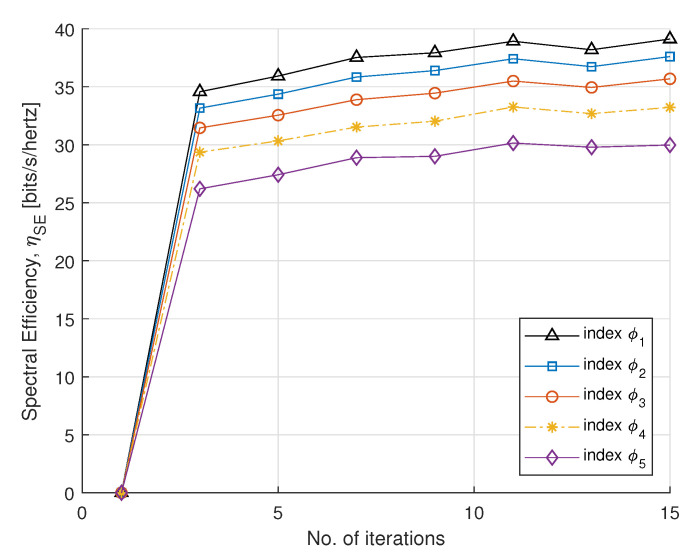
SE vs. number of iterations.

**Figure 4:**
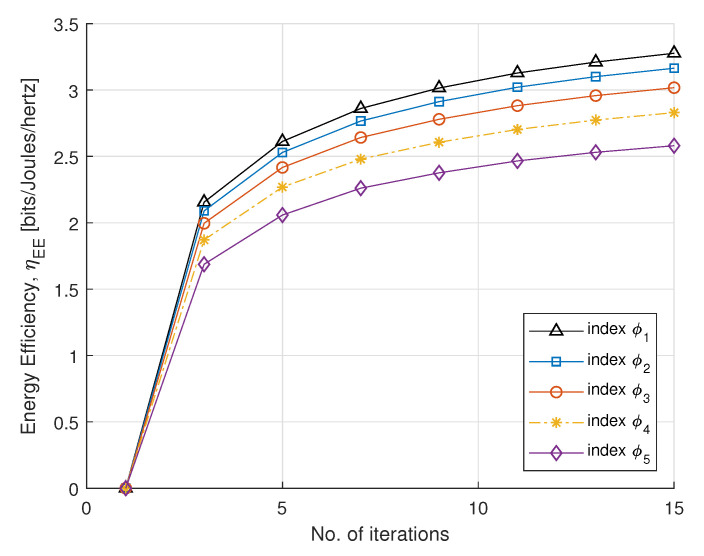
EE vs. number of iterations.

In Figure 3 and Figure 4, we plot SE and EE versus the number of iterations, with fairness index $\phi_{k}$, according to rate constraint in Table 1. The fairness index controls the subcarrier allocation to meet constraints requirements. The minimum EH requirement $E_{min}$ is set to 2 mW. The static circuit power $P_{c}$ is assumed to be 5 W, for a fixed circuit power and we can conclude that EE is nonincreasing for the number of smart meters. For the convergence, the step sizes ($\alpha_{1},\alpha_{2}$, and $\alpha_{3}$) are set to 0.005, which update Lagrange multipliers. For each DA port, the maximum transmit power is set to 30 dBm. It can be observed that EE converges with a few number of iterations. After four iterations, the optimal power maximizes EE and achieves a steady value. This method gives a fairness strategy to control power allocation among PS-SWIPT based smart meters.

**Figure 5:**
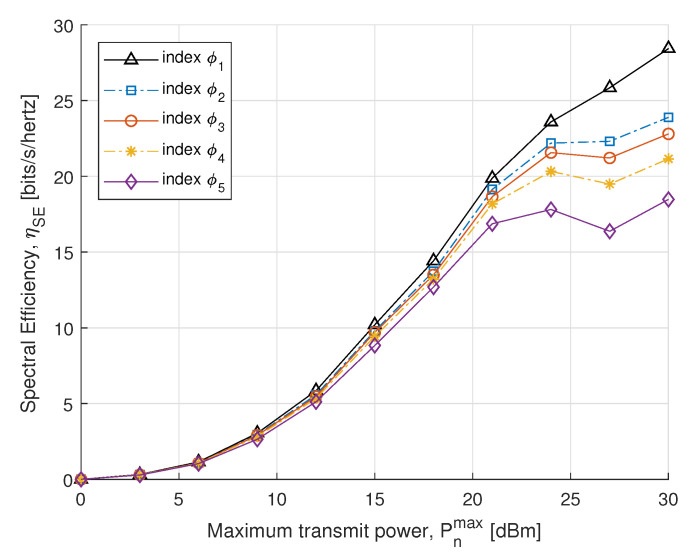
SE vs. transmit power.

**Figure 6:**
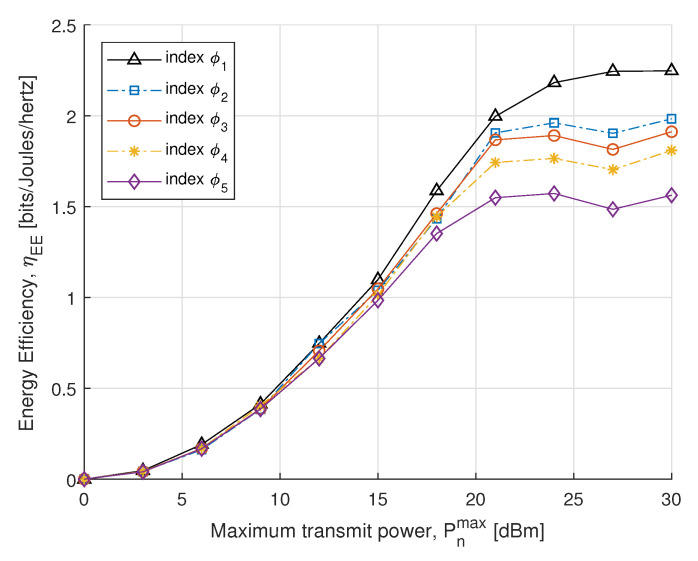
EE vs. transmit power.

In the next simulations, we compare the EE and SE performance for different power constraints with fairness index $\phi_{k}$. In Figure 5, we plot SE with respect to the maximum transmit power constraint. The SE is obtained by the optimal power allocation, which rises dramatically in the maximum allowed transmit power constraint. Initially, no significant difference is found in the case of the small transmit power, whereas after 15 dBm it increases distinctly to achieve the convergence. The result shows that SE of the proposed algorithm converges with an increase in transmit power to meet EH requirements. In Figure 6, the proposed EE maximization algorithm under different maximum transmit power constraint is evaluated. Similar behavior is also reported in [51], where the EE maximization problem approached without SWIPT. It can be seen from Figure 6 that the EE increases sharply for small transmit power constraints and steadily increases for high. Thus, a balance between EE and maximum transmit power constraint can be achieved for the higher transmit power constraint.

**Figure 7:**
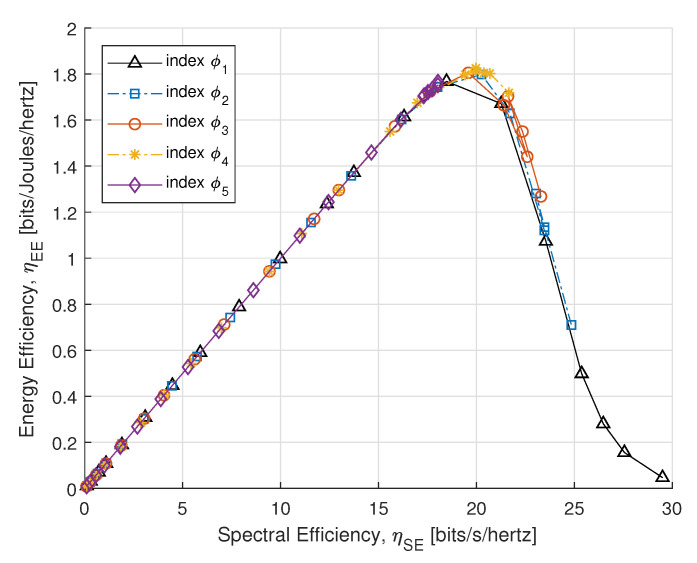
SE vs. EE.

Figure 7 illustrates the SE and EE tradeoff for PS-SWIPT with different rate constraints in Table 1. It shows that the EE increases and approaches a certain peak for a fixed $P_{c}$ and $E_{min}$ acquires no better energy-efficient transmission that is closed to this asymptote.

**Figure 8:**
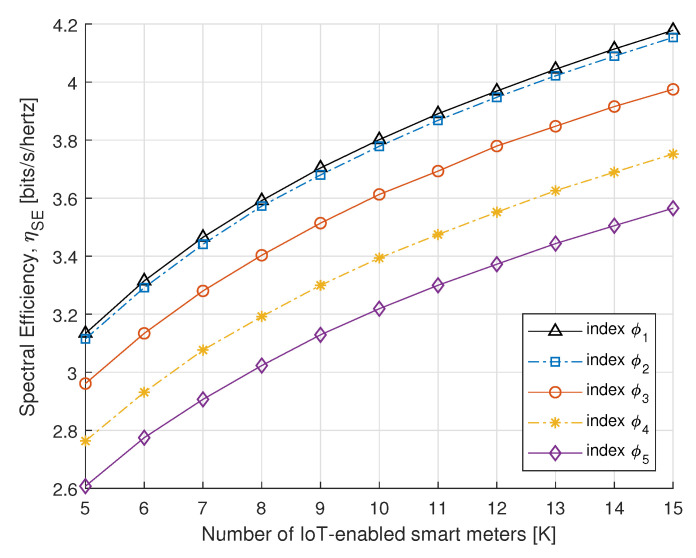
SE vs. number of smart meters.

**Figure 9:**
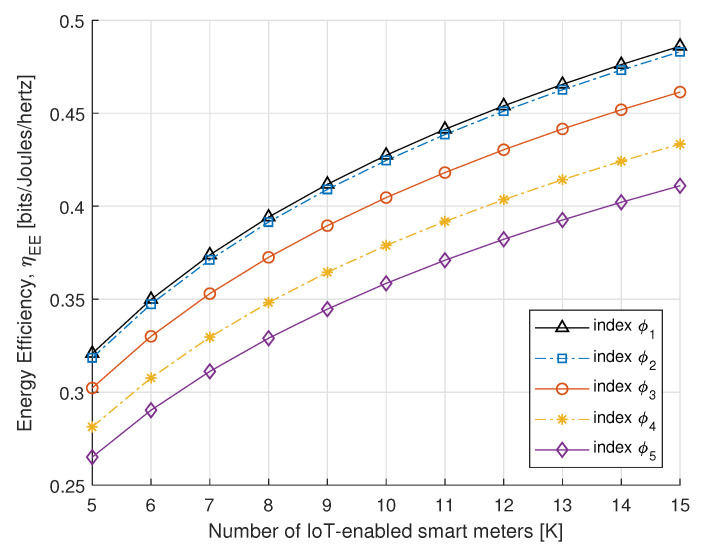
EE vs. number of smart meters.

To further understand the benefits of the proposed algorithm, in Figure 8 and Figure 9, we compare the SE and EE performance of the proposed power allocation with the number of IoT-enabled smart meters. Figure 8 and Figure 9 represent the SE and EE for the PS-SWIPT, as the number of IoT-enabled devices increases with different rate constraint index. These results confirm that sufficient transmit power is available at the DA port if there is an increase in IoT-enabled smart meters.

## 5. Conclusions

This paper addressed the EE maximization problem for IoT-enabled smart meters in DAS-OFDM system. We formulated the EE maximization problem and proposed an algorithm for the optimal transmit power and PS-SWIPT ratio. The algorithm found an optimal solution, which maximizes the EE in the DAS-OFDM system with proportional fairness among IoT-enabled smart meters. The performance of the proposed algorithm demonstrated that the EE converges towards an optimal solution with minimum EH requirements. Furthermore, the numerical results showed that our proposed algorithm achieved the optimal solution with a few iterations. It also confirmed that as the number of IoT-enabled smart meters increases, the EE increases under minimum EH requirements. Therefore, our research will be constructive in solving the difficulty of EE maximization with IoT-enabled PS-SWIPT in the wireless powered smart grid communication networks. Hence, further studies on the current topic are required towards energy-efficient solutions in the uplink case to establish green communication technology for smart grid networks.

## Figures and Tables

**Table 1:** Fairness Rate Constraint.

Fairness index *k*	0	1	2	3	4
$\phi_{1}$ = $\phi_{2}$ = $\phi_{3}$ = $\phi_{4}$ = $\phi_{5}$	$2^{0}$	$2^{1}$	$2^{3}$	$2^{4}$	$2^{5}$
$\phi_{6}$ = $\phi_{7}$ = … = $\phi_{k}$	1	1	1	1	1

**Table 2:** Simulation Paramters.

Parameter	Value
Number of DA ports (N)	5
Number of IoT enabled smart meter devices (K)	15
Number of subcarriers (M)	64
Noise power $\left(\sigma_{z}^{2}\right)$	−104 dBm
Path loss exponent (α)	3.7
Circuit power consumption $\left(P_{c}\right)$	5 W
Shadow fading standard deviation	8 dB
Radius of the cell (R)	1000 m
Maximum transmit power $\left(P_{max}^{n}\right)$	30 dBm
Number of channel realization	$10^{4}$

**Table 3:** Recent Power Allocation Schemes.

Related work	OFDM	DAS	SWIPT
Xu et al. [38]	✓	-	✓
Xu et al. [51]	✓	✓	-
Zhou et al. [52]	✓	-	✓
Yu et al. [53]	✓	✓	-
Our paper	✓	✓	✓

## Data Availability

Not Applicable.
